# Persistent variations in national asthma mortality, hospital admissions and prevalence by socioeconomic status and region in England

**DOI:** 10.1136/thoraxjnl-2017-210714

**Published:** 2018-05-14

**Authors:** Ramyani P Gupta, Mome Mukherjee, Aziz Sheikh, David P Strachan

**Affiliations:** 1 Population Health Research Institute, St George’s, University of London, London, UK; 2 Asthma UK Centre for Applied Research, Centre for Medical Informatics, Usher Institute of Population Health Sciences and Informatics, The University of Edinburgh, Edinburgh, UK

**Keywords:** asthma epidemiology

## Abstract

**Background:**

The UK-wide National Review of Asthma Deaths sought to identify avoidable factors from the high numbers of deaths, but did not examine variation by socioeconomic status (SES) or region.

**Methods:**

We used asthma deaths in England over the period 2002–2015 obtained from national deaths registers, summarised by quintiles of Index of Multiple Deprivation (IMD) and Government Office Region. Emergency asthma admissions were obtained from Hospital Episode Statistics for England 2001–2011. The prevalence of asthma was derived from the Health Survey for England 2010. Associations of mortality, admissions and prevalence with IMD quintile and region were estimated cross-sectionally using incidence rate ratios (IRRs) adjusted for age and sex and, where possible, smoking.

**Results:**

Asthma mortality decreased among more deprived groups at younger ages. Among 5–44 year olds, those in the most deprived quintile, mortality was 19% lower than those in the least deprived quintile (IRR 0.81 (95% CI 0.69 to 0.96). In older adults, this pattern was reversed (45–74 years: IRR 1.37 (1.24–1.52), ≥75 years: IRR 1.30 (1.22–1.39)). In 5–44 year olds the inverse trend with asthma mortality contrasted with large positive associations for admissions (IRR 3.34 (3.30–3.38)) and prevalence of severe symptoms (IRR 2.38 (1.70–3.33)). Prevalence trends remained after adjustment for smoking. IRRs for asthma mortality, admissions and prevalence showed significant heterogeneity between English regions.

**Conclusions:**

Despite asthma mortality, emergency admissions and prevalence decreasing over recent decades, England still experiences significant SES and regional variations. The previously undocumented inverse relation between deprivation and mortality in the young requires further investigation.

**Key messages**

**What is the key question?**

- Do historic variations in asthma outcomes by SES and English region remain despite declines in mortality, admission and prevalence rates?

**What is the bottom line?**

- Large and highly significant differences in both regional and SES patterns remain and the latter show surprisingly anomalous results in the young.

**Why read on?**

- The UK National Review of Asthma Deaths has highlighted the importance of psychosocial factors in asthma deaths and we have confirmed the contributory role of relative deprivation. Although severe asthma prevalence and emergency hospital admission rates for asthma were much higher in less affluent areas, asthma mortality among children and younger adults was more common in more affluent areas. Previously reported regional variations in asthma mortality within England persist but correlate poorly with the regional prevalence of severe asthma.

## Background

The UK has among the highest asthma mortality rates of high-income countries globally in younger age-groups (those aged 5–34 years), the highest asthma admission rates in Europe and the highest rates of asthma symptoms globally in children.^1^ The National Review of Asthma Deaths (NRAD), a confidential review of asthma deaths, found that despite improved asthma care in recent decades many deaths were still preventable.^2^ Variations in asthma prevalence, hospitalisation and death by socioeconomic status (SES)^3 4^ and region^5 6^ have been highlighted previously, but covered short time periods or age-ranges or were undertaken several years ago.

The independent impact of SES on premature mortality has been compared with that from physical inactivity and smoking^7^ though it is acknowledged that there are complex relationships between these and other mortality risk factors. That SES has a significant role in reported asthma symptoms, doctor-diagnosis, hospital admission rates and mortality has long been accepted,^3 8 9^ but there are relatively few conflicting accounts of its effects in children and younger adults.^4 10 11^

In the light of NRAD, which did not address SES or geographical trends, we updated and extended earlier reviews^3 4 6^ to consider trends by deprivation and region using recent asthma prevalence, admission and death data in England spanning the last 15 years.

## Methods

As a recent extensive review of asthma burden in the UK illustrated, harmonisation of asthma definitions was not possible across the UK and not all outcomes were available by SES.^12^ We have therefore confined this investigation to England, which accounts for 85% of the UK population. All analyses were undertaken in Stata 12.^13^

### Deaths and populations

The numbers of registered deaths in England with underlying cause of asthma (International Classification of Diseases Version 10 (ICD-10) J45 and J46) recorded on the death certificate were obtained from the Office of National Statistics (ONS).^14^ Deaths were included from 2002, after ICD-10 was introduced into the UK mortality data, until 2015, the latest available year. Individual level data included postcodes, which were matched to English Index of Multiple Deprivation (IMD) 2010 ranks.^15^ Deaths by quintile of IMD were derived and ranged from 1 (least deprived) to 5 (most deprived). Death data included English county, from which the nine Government Office Regions (GOR) of England were derived. Mid-year population estimates were obtained from ONS by year, 5-year age group, sex, English IMD 2015 and GOR.^14^

Indirect standardisation (the calculation of Standardised Event Ratios, SERs) was used to compare asthma mortality by IMD quintile and GOR in England. An SER of 100 represents the England average age-standardised ratio.

Incidence rate ratios (IRR) by IMD and GOR were calculated using Poisson regression on IMD quintile and GOR as categorical variables adjusting for age and sex. Trends in SES were fitted using linear regressions and the linear model fit tested using likelihood ratio tests.

Results for IMD were stratified into broad age-ranges (5–44, 45–74 and 75 years and over). The youngest group was the one for whom asthma was unlikely to be confused with chronic obstructive pulmonary disease (COPD), found in older people, or with other respiratory conditions of the very young. The oldest group were excluded from NRAD partly because ‘resources did not permit’,^2^ but included here for completeness. Regional results were presented for all ages above 5 years; age-stratified results would lead to less statistical reliability.

### Emergency hospital admissions

Numbers of emergency admissions with a primary diagnosis of asthma (ICD-10 J45 and J46) by age, sex, IMD 2004 quintile were obtained from Hospital Episode Statistics (HES) for England for calendar years 2001-2011.^16^ We also obtained data from an analysis of England 2008–12 admissions^5^ which included information on age, sex and GOR. SERs by IMD quintile and GOR were calculated using national population denominators and age-sex-adjusted IRRs were derived.

### Health Survey for England

The prevalence of asthma symptoms, diagnosis and treatment were derived from the Health Survey for England (HSE) 2010, the latest of these annual surveys to include a detailed respiratory questionnaire. HSE is a large, nationally representative health examination survey of households in England in 2010. Trained interviewers surveyed 14 112 adults and children of all ages with response rates between 66% and 70%.^17^ Two measures of prevalence were used, the prevalence of clinician-diagnosed-and-treated asthma and recent severe asthma, as detailed in the footnotes to table 1. IMD 2007 quintile, GOR and non-response weights were included in HSE 2010. Age-sex-adjusted IRRs were calculated to compare with those for mortality and admissions. Additional adjustment for smoking status was possible in HSE (never smoked versus currently/ever smoked cigarettes). SERs were derived by IMD quintile by the broad age-bands and by GOR for all ages due to small numbers.

**Table 1:** Association of Index of Multiple Deprivation (IMD) quintile with asthma outcomes (incidence rate ratio (IRR) with 95% CI), adjusting for age and sex, stratified by age-band: (A) mortality, England 2002–2015; (B) emergency admissions, England 2001–2011; (C) prevalence of clinician-diagnosed-and-treated asthma in the last 12 months, England 2010*; (D) prevalence of recent severe asthma symptoms*, England 2010.

(A) Mortality	5–44 years				45–74 years				75 years and over			
IMD	N deaths	IRR	95% CI		N deaths	IRR	95% CI		N deaths	IRR	95% CI	
Least deprived 1	263	1.00	Reference		765	1.00	Reference		1847	1.00	Reference	
2	291	0.96	0.81	1.13	767	1.07	0.97	1.18	1751	0.94	0.88	1.00
3	244	0.85	0.72	1.02	768	1.11	1.00	1.22	1750	0.95	0.89	1.02
4	297	0.85	0.72	1.01	783	1.28	1.16	1.41	1854	1.14	1.07	1.22
5	275	0.81	0.69	0.96	751	1.37	1.24	1.52	1866	1.30	1.22	1.39
Linear trend for IMD		0.95	0.91	0.99		1.08	1.06	1.11		1.08	1.06	1.09
(P value)		0.006				<0.001				<0.001		

(B) Admissions	5–44 years				45–74 years				75 years and over			
IMD	N admissions	IRR	95% CI		N admissions	IRR	95% CI		N admissions	IRR	95% CI	
Least deprived 1	40 428	1.00	Reference		18 309	1.00	Reference		7201	1.00	Reference	
2	47 402	1.22	1.20	1.23	23 433	1.17	1.15	1.19	8745	1.09	1.06	1.13
3	59 025	1.64	1.62	1.66	27 775	1.23	1.21	1.25	10 044	1.13	1.10	1.17
4	79 155	2.28	2.25	2.31	35 044	1.47	1.45	1.50	10 991	1.23	1.20	1.27
5	1 13 570	3.34	3.30	3.38	49 385	2.01	1.98	2.05	12 370	1.43	1.39	1.47
Linear trend for IMD		1.37	1.37	1.37		1.19	1.19	1.20		1.09	1.08	1.10
(P value)		<0.001				<0.001				<0.001		

(C) Diagnosis	5–44 years				45–74 years				75 years and over			
IMD	N diagnosed	IRR	95% CI		N diagnosed	IRR	95% CI		N diagnosed	IRR	95% CI	
Least deprived 1	123	1.00	Reference		56	1.00	Reference		14	1.00	Reference	
2	136	1.21	0.96	1.54	59	1.41	1.00	1.99	16	1.06	0.54	2.05
3	147	1.31	1.04	1.66	66	1.58	1.13	2.22	13	1.17	0.59	2.32
4	157	1.27	1.01	1.60	87	1.99	1.44	2.75	18	2.06	1.11	3.83
5	184	1.38	1.10	1.73	70	2.14	1.53	2.98	10	1.38	0.67	2.84
Linear trend for IMD		1.07	1.02	1.12		1.20	1.12	1.29		1.16	1.00	1.34
(P value)		0.007				< 0.001				0.06		

(D) Symptoms	5–44 years				45–74 years				75 years and over			
IMD	N severe symptoms	IRR	95% CI		N severe symptoms	IRR	95% CI		N severe symptoms	IRR	95% CI	
Least deprived 1	47	1.00	Reference		36	1.00	Reference		14	1.00	Reference	
2	59	1.47	1.01	2.13	37	1.36	0.89	2.10	14	0.99	0.51	1.89
3	71	1.77	1.23	2.53	53	1.92	1.28	2.88	11	0.87	0.43	1.78
4	94	2.00	1.42	2.84	65	2.35	1.59	3.48	20	2.12	1.16	3.86
5	113	2.38	1.70	3.33	79	3.85	2.65	5.59	8	1.10	0.52	2.32
Linear trend for IMD		1.22	1.13	1.31		1.39	1.28	1.51		1.13	0.97	1.32
(P value)		<0.001				< 0.001				0.12		

*Clinician-diagnosed-and-treated asthma; a combination of the questions: ‘Did a doctor or nurse ever tell you that you had asthma?’ AND “Over the last 12 months, have you used an inhaler, puffer or nebuliser prescribed by a doctor to treat your asthma, wheezing or whistling, or difficulty in breathing?”. Recent severe asthma; a combination of any of the following: in the last 12 months has had sleep disturbed one or more nights per week due to wheezing/whistling in chest, has found in the last 12 months chest wheezing ±whistling interfered with normal activities ‘quite a bit’ or ‘a lot’, has experienced symptoms of asthma ‘every day’ or ‘most days’, had difficulty sleeping one or more days due to usual asthma symptoms in the last week or had usual asthma symptoms during the day one or more days in the last week

### Reporting

The STROBE statement for cross-sectional studies was used to guide reporting of results.

## Results

In England, there were 14 830 recorded asthma deaths between 2002 and 2015 of which 68% occurred in females; there were 1424 in 5–44 year olds (255 in 5–14 years), 3993 in 45–74 year olds and 9388 in those over 75 years. There were 542 877 emergency asthma admissions over the age of 5 years from 2001 to 2011, of which 60% occurred in females. In HSE 2010, of 12 077 people over the age of 5 years (the total number after weighting for age-sex representation and non-response), 1156 reported having been diagnosed and treated for asthma (51% female) and 721 reported severe symptoms of asthma in the last year (52% female).

### Variation by deprivation quintile

IMD was matched for 96% of deaths, 99% of admissions and 100% of survey respondents. SERs for quintiles of IMD showed distinct patterns by outcome, age-band and sex. Except for mortality in ages 5–44 years, there were broadly increased event ratios with increasing deprivation for all age-groups for mortality, hospital admissions and severe symptoms. All showed statistically significant linear trends except for symptoms in age 75 years and over, where smaller numbers were surveyed. By contrast, in 5–44 year olds there was a pattern of decreasing asthma mortality in more deprived areas. Clinician-diagnosed-and-treated asthma showed mixed results across age and sex groups with a clear increase with deprivation only in the 45–74 years group.

**Figure 1:**
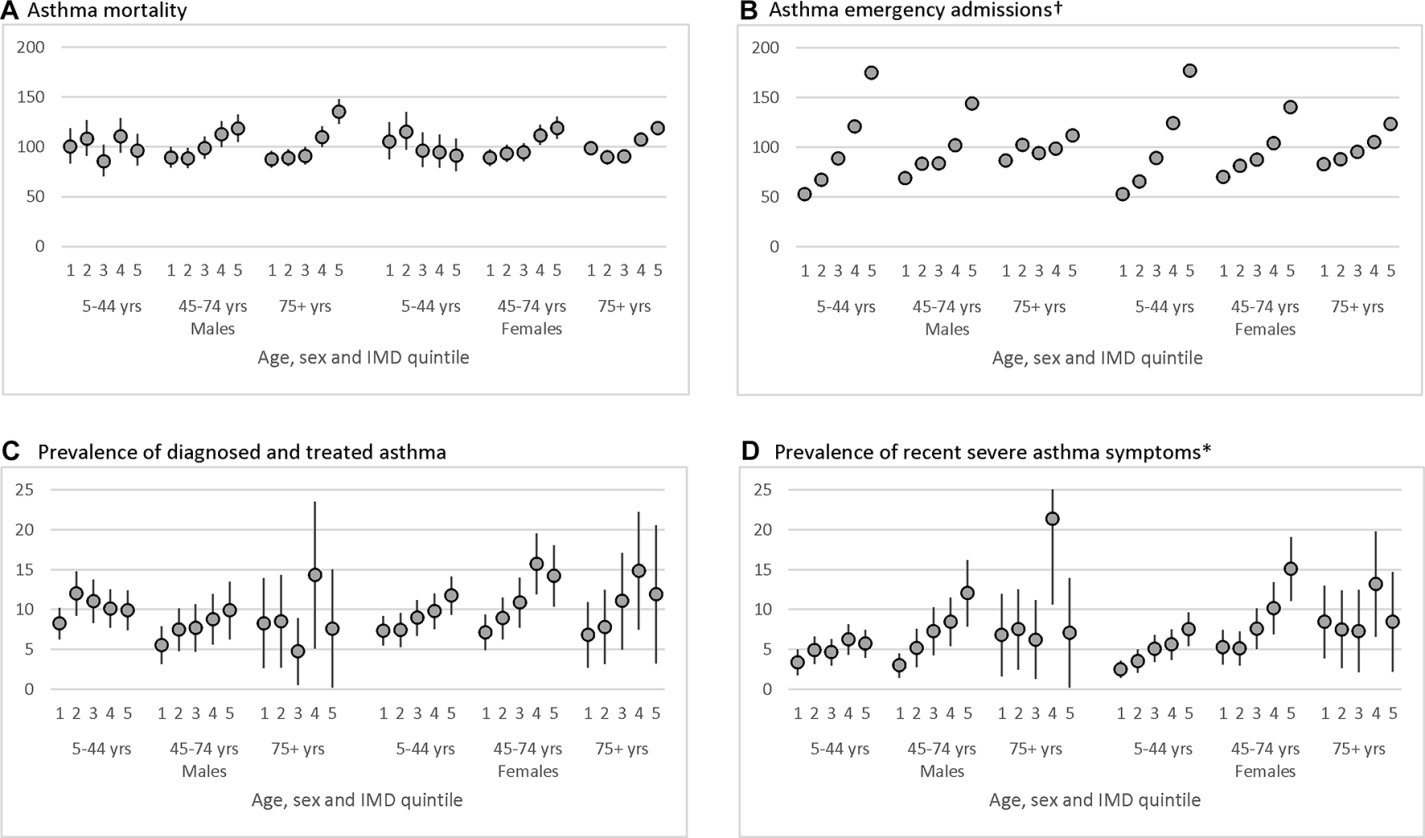
Socioeconomic status variations in asthma outcomes in England, standardised event ratios by Index of Multiple Deprivation (IMD; 1 is least deprived), sex and age-band, with 95% CI bars: (A) mortality 2002–2015, Office for National Statistics mortality; (B) emergency admissions 2001–2011, Hospital Episode Statistics; (C) prevalence of clinician-diagnosed and treated asthma 2010*, Health Survey for England 2010; (D) prevalence of recent severe asthma 2010*, Health Survey for England 2010. Footnotes. †95% CI too small to be visible. *Clinician-diagnosed-and-treated asthma; a combination of the questions: ‘Did a doctor or nurse ever tell you that you had asthma?’ AND ‘Over the last 12 months, have you used an inhaler, puffer or nebuliser prescribed by a doctor to treat your asthma, wheezing or whistling, or difficulty in breathing?’. Recent severe asthma; a combination of any of the following: in the last 12 months has had sleep disturbed one or more nights per week due to wheezing/whistling in chest, has found in the last 12 months chest wheezing ±whistling interfered with normal activities ‘quite a bit’ or ‘a lot’, has experienced symptoms of asthma ‘every day’ or ‘most days’, had difficulty sleeping one or more days due to usual asthma symptoms in the last week or had usual asthma symptoms during the day one or more days in the last week. Presented as simple prevalences (%) due to small numbers.

In females aged 5–44 years, SERs for mortality broadly decreased with increasing deprivation while in males the pattern was more mixed (figure 1A). There was an average 5% (95% CI 1% to 9%) fall in age-sex-adjusted IRRs for asthma mortality in this youngest age group from the least deprived to the most deprived (test for trend from linear regression, P=0.006, table 1A). In those aged 45–74 years, the trend ran in the opposite direction with SERs rising from the least deprived to the most deprived quintile in both males and females. There was a significant 8% increase (95% CI 6% to 11%) in IRRs across increasing IMD quintile. In those over 75 years, there was a similar rise in mortality with increasing deprivation, but with a U-shaped pattern. These mortality differentials were broadly consistent over time, but numbers were small per year in the youngest age-band precluding detailed analysis. Average mortality rates in the 5–44 year olds were higher in the most affluent areas in each of 2002–05, 2006–2010 and 2011–2015 (7.0, 6.2 and 5.2 per million for the least deprived quintile compared with 6.6, 5.3 and 3.8 in the most deprived quintile). In contrast, among the 45–74 year olds mortality rates were lower in the least deprived quintile compared with the highest in each of these three time periods (20.8, 15.5 and 11.9 compared with 27.9, 19.8 and 17.5).

Neither the IMD trend in emergency asthma admissions (figure 1B) nor in prevalence of asthma (figure 1C, D) showed these same age-specific differences. In the<45 years age group there was a more than threefold increase in asthma admissions in the most deprived IMD quintile compared with the least (IRR=3.34, 3.30–3.38, table 1B), a doubling in the 45–74 year olds (IRR=2.01,1.98–2.05) and a 43% increase in the oldest age-band. Differences in the prevalence of diagnosed-and-treated asthma as well as severe asthma showed similar patterns though these were smaller than that for admissions and greater than that for mortality (table 1C, D). Interpretation of the relative prevalence in the oldest age group was hindered by the smaller numbers surveyed. Adjusting the prevalences for smoking, possible only in the survey data, led to a small attenuation in the otherwise similar pattern of rates by IMD (online supplementary table 1).

10.1136/thoraxjnl-2017-210714.supp1 Supplementary file 1

### Variation by English region

**Figure 2:**
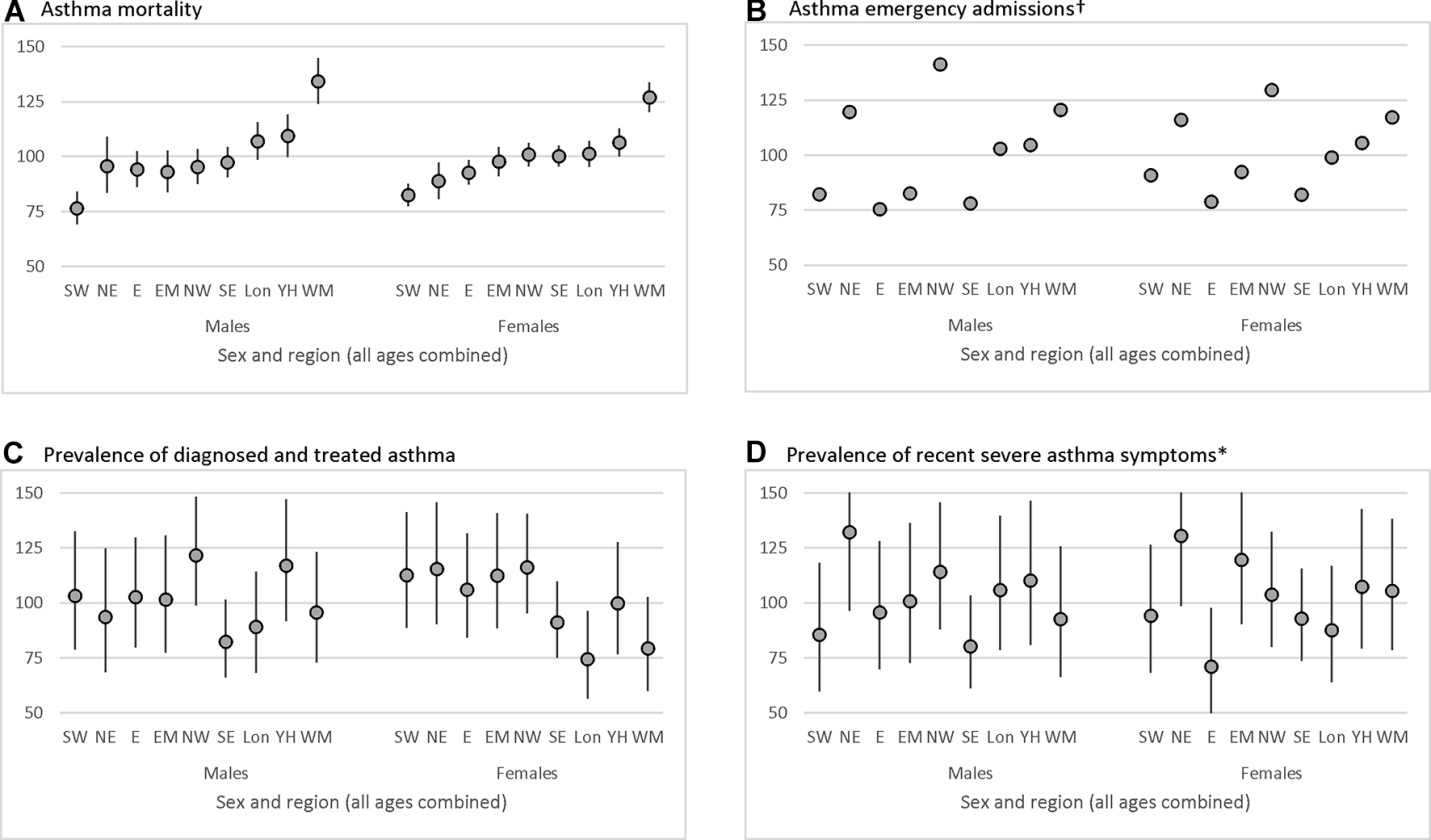
Regional variations in asthma outcomes in England, standardised event ratios by English Government Office Region and sex, with 95% CI bars, ranked by the Standard Events Ratios (SERs) for mortality in males and females combined: (A) mortality 2002–2015, ONS mortality; (B) emergency admissions 2008–12, Hospital Episode Statistics; (C) prevalence of clinician-diagnosed-and-treated asthma 2010, Health Survey for England 2010; (D) prevalence of recent severe asthma 2010*, Health Survey for England 2010. Footnotes. †95% CI too small to be visible. *Clinician-diagnosed-and-treated asthma; a combination of the questions: ‘Did a doctor or nurse ever tell you that you had asthma?’ AND ‘Over the last 12 months, have you used an inhaler, puffer or nebuliser prescribed by a doctor to treat your asthma, wheezing or whistling, or difficulty in breathing?’. Recent severe asthma; a combination of any of the following: in the last 12 months has had sleep disturbed one or more nights per week due to wheezing/whistling in chest, has found in the last 12 months chest wheezing ±whistling interfered with normal activities ‘quite a bit’ or ‘a lot’, has experienced symptoms of asthma ‘every day’ or ‘most days’, had difficulty sleeping one or more days due to usual asthma symptoms in the last week or had usual asthma symptoms during the day one or more days in the last week. Government Office Regions (GOR), SW South West, NE North East, E East of England, EM East Midlands, NW North West, Lon London, YH Yorkshire and Humberside, WM West Midlands.

GOR was available for all deaths, admissions and survey respondents and showed significant regional variations for all outcomes. Mortality rates were highest in the West Midlands for both males and females (figure 2A), about a third higher than the England average (males SER=134, 95% CI 124 to 145; females SER=127, 120–134). Age-sex-adjusted IRRs reflected these patterns and showed significant heterogeneity by region (P<0.001, online supplement table 2).

The highest emergency admission rates occurred in the North West (figure 2B, males SER=141, 139–143, females SER=129, 128–131). Again the adjusted IRR reflected these patterns and showed significant regional heterogeneity (P<0.001, online supplementary table S2).

There were slightly smaller differences in SERs for the prevalence of clinician-diagnosed-and-treated asthma, from the highest in the North West to the lowest in South East. Severe asthma symptoms SERs were highest in the North East and lowest in the South East for males and East of England for females. Despite the smaller numbers in the survey sample, there was significant heterogeneity in the adjusted IRRs of prevalence measures between regions (P=0.005 for diagnosed-and-treated asthma and P=0.04 for severe asthma).

Although SERs for mortality and admission were highest in the West Midlands, prevalence of both diagnosed asthma and severe symptoms were lower than the national average there. In general, there was little congruence between outcomes by GOR (all correlations r<|0.43| online supplementary table 3). The exception to this was a correlation between those reporting severe symptoms and hospital admissions in males and females combined of 0.60 (P=0.01 for Spearman test of independence).

## Discussion

### Statement of principal findings

We have shown that despite well-known recent declines in national asthma mortality and hospital admission rates,^18^ significant differences by SES and region remain. We have also highlighted an unusual and unexpected pattern of a modest but significant decrease in deaths from asthma with increasing levels of relative deprivation in children and adults<45 years.

This was not consistent with the trend in national emergency admissions, where there was a trebling with increasing deprivation, nor in self-reported levels of clinically significant asthma, where there was a doubling in prevalence, in the same age group. For older age groups, in each of these outcomes there remained a clear trend of increasing asthma deaths, admissions and prevalence with greater relative deprivation. The IMD trends in measures of asthma prevalence were attenuated, but remained, after adjustment for smoking.

Relative differences between the lowest and highest regional rates ranged between 1.5 to 1.9 times for the outcomes investigated here. Although the West Midlands still has among the highest rates for both asthma mortality and emergency admissions in England, regional patterns of admissions and prevalence across England do not generally reflect those for mortality.

### Strengths and weaknesses

We have used national datasets over a number of overlapping years to assess asthma patterns by SES and region though we were unable to link these to compile a longitudinal dataset. UK deaths statistics have excellent completion rates^14^ though NRAD’s detailed investigation into cause of death showed a drop in the accuracy of deaths coding with increasing age.^2^ In those under 45 years, the NRAD Panel agreed that clinical findings matched asthma as the underlying cause in over 90% of cases. The youngest death in that study was aged 4 suggesting that our 5–44 years group has the most accurate asthma mortality coding of all age-groups. This proportion fell to 60–85% in those aged 45–74 years and under 50% in those sampled over 75 years when mis-classification with COPD and other comorbidities increase. However, the percentage of deaths with a mention of asthma which have asthma as the underlying cause has not changed appreciably over time^19^ and there has to our knowledge been no suggestion of any association of deaths coding with SES or region. Low numbers of asthma deaths in the youngest age group prevented more detailed breakdowns by age or other risk factors to investigate further. HES data have high completeness nationally and a recent review found a median 80% accuracy in diagnostic coding.^20^ Neither deaths nor admissions data include information on smoking. Prevalence from HSE is self-reported, but this nationally representative survey has good response rates from a large sample and uses validated prevalence questions.^17^ In contrast to the surveys used in earlier reviews,^6^ for the HSE 2010 non-response weights were calculated adjusting for age, sex, Strategic Health Authority, household type and the social class of the household reference person.

IMD is a widely used, regularly updated, area-based, multifaceted measure of SES with seven components including health, employment, income and education. It applies beyond working-aged groups, reflects households rather than individuals and may be less prone to reverse-causation than purely income or employment based measures. Although the IMD measures presented are from different years for some outcomes, those for 2004, 2007, 2010 and 2015 employed here use the same methodology and were designed to be broadly comparable in terms of relative deprivation.^21^ We found 3.6% missing IMD quintile in the mortality data, but there was little difference by age-group (3.8% 5–44y, 4.0% 45–74y and 3.4% 75+). We have no reason to believe IMD coding is less accurate in younger age groups though deprivation measures tend to improve with age. This does not explain however the inverse relationship seen for mortality compared with that for admissions or prevalence.

### Interpretation in the light of previous research

Previous UK studies used social class (SC I to V) as a measure of SES.^4 5 8 9^ This was derived from the occupation of the individual or head of household but could not be applied to those over working age. Higgins *et al* 4 found a similar pattern to this investigation: little difference in mortality between SC in males aged 5–34 years (England and Wales 1979–87), but in men aged 35–64 years there were 36% more deaths in manual occupations compared with non-manual. Two-thirds of records for women were missing individual SC and in those remaining there was no clear trend. Jones *et al* 8 considered a broader age range and found that in those aged 5–55 years (from deaths in 401 local authority districts in England in 1988–92) asthma mortality was strongly associated with the proportion of households where the head was SC most deprived (RR=1.61, 95% CI 1.12 to 2.33). National mortality records (all-ages SERs, England and Wales 1991–93) showed a four-fold increase in asthma deaths between those in the most and least deprived SC.^9^

The more recent mortality rates among older adults presented here show higher rates in more deprived areas, but the observation of a significant trend in the opposite direction among children and younger adults may reveal an unexpected phenomenon. Among 5–44 year-olds, the prevalence of diagnosed-and-treated asthma varied little with area deprivation in contrast to a doubling in prevalence of severe symptoms (table 1). While this might suggest small differences in asthma labelling by SES (even after adjustment for smoking), most likely to be made by primary care physicians, the mortality results for this age-group are unlikely to be entirely attributable to diagnostic or labelling artefact. Since severe asthma symptoms and hospital admissions for asthma in the under-45s were less common in the more affluent areas it is unlikely that the higher mortality rate in this group is due to poorer long-term control of asthma symptoms. Indeed, one would expect that compliance with prescribed treatment would be better in these areas than in more deprived areas. We were unable to assess trends by SES in general practice data, but two studies which have done this showed higher prevalence and incidence of asthma consultations in more deprived areas,^5^ a socioeconomic trend which has been found even among non-smokers.^9^ SES measures from routine data are difficult to harmonise internationally so comparable SES measures between the UK and elsewhere are rare and we are not aware of any for asthma risk factors. Nor are we aware of any analyses of SES time-trends for asthma, again probably due to the difficulties in comparisons over time, though our own analysis shows consistently higher mortality rates in the most deprived quintile compared with the least deprived quintile (online supplementary figure 1).

Significant differences in asthma mortality, admissions and prevalence by English region have been noted in the past.^4 6^ The weak correlations found between regional all-ages asthma prevalence, admissions and mortality in the early 1990s^6^ match our results. This is perhaps unsurprising as the highest admission rates are in the young whereas the highest mortality rates occur in the elderly with prevalence more stable over the life course. We found that large regional differences persist despite the introduction, in the late 1990s, and the development, over several decades, of national treatment guidelines by the National Institute for Health and Care Excellence and BTS/SIGN as well as several years’ of incentive payments for asthma treatment by the Quality and Outcomes Framework programme. This concurs with GP reports of people ‘ever diagnosed’ with asthma showing wide variations across England in 2012.^5^ An analysis of 24 European countries found a very similar correlation (r=0.44, P=0.03) between them in age-standardised mortality and admissions for asthma.^1^ A comparison of mortality, admissions and prevalence of wheeze in children^22^ similarly found a high correlation between severe wheeze and admissions in 13–14 year olds (r=0.73, P=0.003) in 14 countries though little correlation between other outcomes.

Regional variations in asthma mortality in the UK have been shown to reflect geographical differences in access to and quality of healthcare services^6^ as well as socioeconomic factors.^8^ Information on more specific exposures such as smoking and ethnicity was not available in the national mortality and admissions data, but using the prevalence data we were able to confirm the observation of Hansell *et al* 6 8 that regional differences in asthma prevalence and severity were not altered greatly by adjustment for smoking. An analysis of deaths from all causes in England^23^ found that IMD accounted for a large proportion of the geographical variation in age-adjusted mortality and the independent contribution of regional differences was small in both sexes.

### Interpretation and future research

Stringini *et al* 7 have estimated the rate-ratio of low compared with high SES on non-cardiovascular disease, non-cancer mortality to be 1.45 (95% CI 1.35 to 1.56) after adjustment for age, sex and ethnicity and 1.25 (95% CI 1.17 to 1.33) when further adjusted for high alcohol intake, physical inactivity, current smoking, hypertension, diabetes and obesity. They acknowledge that each of these may be interrelated, together with specific components of SES including low income, education levels and occupational position. Rona^3^ suggested the following possible reasons for SES as a risk factor in asthma: an aetiological factor; an exacerbating factor; a determinant of the quality of care; a contributor of psychosocial behaviour which impacts on the management and prognosis of the condition or as a component in asthma labelling. More specifically, the Global Atlas of Asthma^24^ suggests that low SES families are more likely to be exposed to environmental pollutants (eg, particles from diesel), indoor allergens (eg, mould and dust) and other respiratory irritants (eg, tobacco smoke) that adversely affect asthma as well as being more likely to be exposed to psychosocial stressors (eg, food, housing and financial insecurity, social marginalisation and community or domestic violence). NRAD^2^ found psychosocial factors contributed to 26% (51/195) of UK asthma deaths of all ages, with similar proportions among deaths under 10 (30%) and aged 10–19 (22%). ‘Psychological factors’ contributed to 16% of deaths and ‘social factors’ to 15% of deaths. There are well-recognised occupational causes of asthma associated with lower SES jobs (eg, bakers, farmers, painters, welders, woodworkers). These, as with most factors in the literature, help explain the increased asthma in lower SES groups seen in most outcomes and age-groups here and elsewhere.

There are few examples however of risk factors of any type for higher SES groups that might account for our mortality findings in the 5–44 year olds. The Global Atlas of Asthma suggests ‘community vitality/collective efficacy, increased maternal-child interaction, and effective utilisation of psychological coping strategies’ have positive effects on asthma outcomes and it could be that some of these form mitigating factors in lower SES groups. There are also a few occupational causes of asthma associated with higher SES (eg, latex in healthcare workers, aldehydes in dentists, animal excreta in laboratory researchers). It is not clear how much each of these might contribute to the inverse pattern seen here in younger deaths or if other factors might account for it. Higgins and Britton were not able to provide an explanation found for the similar pattern found in males in earlier decades.^4^ A study of mortality in England in the late 1990s^25^ found slightly higher mortality rates from ‘major causes’ in teenage boys in the least deprived quintile of IMD compared with the most deprived and almost equal rates in teenage girls. This category included lower respiratory diseases, cancers, circulatory disease and others (ICD9 1–239 390–519) but no reasons for this pattern were offered nor were more detailed causes investigated. A study comparing mortality with a census question on long-term illness or disability^26^ found SES all-cause mortality differentials were lowest in the teenage years and early 20s, but also found a similar pattern for self-reported ‘limiting long-term illness’, in contrast to our findings here for asthma. This suggests a disease-specific component to the difference between mortality and morbidity SES patterns for asthma.

One speculative explanation for the anomalous socioeconomic trend in younger asthma deaths is that in more affluent areas there is a higher prevalence of a less predictable (more ‘brittle’) form of asthma which occasionally progresses rapidly to a fatal outcome despite apparently adequate long-term symptomatic control. The higher prevalence of atopy and allergies in higher SES groups may also be a factor acting together with brittle asthma or in addition to it and it is possible that some of these cases are included within deaths coded as asthma.

Asthma poses a large healthcare burden in England costing the public sector an estimated £900 million^12^ and many deaths are avoidable with better care.^2^ Despite falling asthma mortality over time and decades of improvements in asthma management, the UK still suffers among the highest global mortality rates in the young^1^ and significant differences by SES and region remain across England, despite national guidelines and a health service free at the point of contact. These differences are even wider in emergency hospital admissions where there were more than three times as many emergency admissions in the poorest among the youngest and twice as many admissions and cases of severe symptoms among the poorest in older age groups.

These results should be monitored in England and, where possible, tested in other countries using existing international health studies. Greater numbers of deaths from longer English or UK time trends could be sought to investigate age-specific mortality-SES differentials within the 5–44 year old age-band. The relationships between prevalence of symptoms, consultations in general practice, emergency admission to hospital and mortality should be examined further and the reason for the inverse relation of mortality with IMD in those aged 5–44 years in relation to other age groups and other outcomes needs explanation as this is unlikely to be due to data artefact. Such analyses could use general practice databases linked to admissions and mortality which would enable more complete allowance than possible here, for confounding by smoking and other risk factors. More detailed clinical information could identify a distinct and troublesome asthma phenotype which has become more common in England in recent years, particularly in more affluent areas, as well as helping identify the role of concurrent allergic disease or any overlap with COPD in older age groups.
