# Supplementary material for: Persistent variations in national asthma mortality, hospital admissions and prevalence by socioeconomic status and region in England
Source: Thorax. 2018 May 14;73(8):706–12. doi: 10.1136/thoraxjnl-2017-210714 (PMC6204968; doi:10.1136/thoraxjnl-2017-210714)

ONLINE SUPPLEMENT

Table 1. Effect of IMD quintile on prevalence of asthma outcomes (Incidence Rate Ratios), adjusting for age, sex and smoking cigarettes[1], stratified by broad age band. England 2010: (b) prevalence of clinician-diagnosed and treated asthma in the last 12 months (b) prevalence of recent severe asthma symptoms in the last 12 months[2].

(a) Diagnosis	5-44 years				45-74 years				75 years and over			
IMD	N diagnosed	IRR	95% CI		N diagnosed	IRR	95% CI		N diagnosed	IRR	95% CI	
Least deprived 1	123	1.00	Reference		56	1.00			14	1.00		
2	136	1.14	0.79	1.65	59	1.40	0.99	1.97	16	1.06	0.54	2.05
3	147	1.29	0.91	1.83	66	1.52	1.08	2.14	13	1.13	0.57	2.24
4	157	1.10	0.77	1.58	87	1.91	1.38	2.64	18	1.94	1.04	3.61
5	184	1.36	0.97	1.90	70	2.03	1.45	2.84	10	1.28	0.62	2.65
Linear trend for IMD		1.06	0.98	1.14		1.19	1.10	1.28		1.13	0.97	1.32
(p-value)		0.13				<0.001				0.11		
**(b) Symptoms**	5-44 years				45-74 years				75 years and over			
IMD	N severe symptoms[2]	IRR	95% CI		N severe symptoms[2]	IRR	95% CI		N severe symptoms[2]	IRR	95% CI	
Least deprived 1	47	1.00	Reference		36	1.00			14	1.00		
2	59	1.14	0.64	2.02	37	1.34	0.87	2.06	14	0.98	0.51	1.88
3	71	1.75	1.05	2.92	53	1.79	1.19	2.68	11	0.85	0.41	1.73
4	94	1.78	1.07	2.95	65	2.17	1.47	3.21	20	1.99	1.09	3.63
5	113	2.22	1.37	3.60	79	3.50	2.40	5.08	8	1.02	0.48	2.17
Linear trend for IMD		1.22	1.10	1.35		1.36	1.25	1.48		1.11	0.95	1.29
(p-value)		<0.001				<0.001				0.2		

1 Currently smoking or ever smoked cigarettes compared with never smoked cigarettes.

2 Frequent symptoms, interference with normal activities or sleep disturbance in the last year.

Table 2. Regional variation (Incidence Rate Ratios), adjusted for age and sex: mortality (England 2002-2015), admissions (England 2008-2012), treated asthma (England 2010), severe asthma (England 2010)

	Mortality				Emergency admissions				Treated asthma				Severe asthma			
Government Office Region of England	N	IRR			N	IRR	95% CI		N	IRR	95% CI		N	IRR	95% CI	
South East	2,555	Reference			39,961	Reference			201	Reference			140	Reference		
North East	675	0.88	0.81	0.96	17,338	1.45	1.42	1.47	118	1.23	0.98	1.55	101	1.49	1.16	1.93
North West	1,947	0.98	0.92	1.03	54,620	1.66	1.64	1.68	205	1.37	1.13	1.67	129	1.24	0.97	1.57
Yorkshire & Humber	1,583	1.06	1.00	1.13	31,968	1.29	1.27	1.31	137	1.25	1.00	1.55	95	1.24	0.96	1.61
East Midlands	1,233	0.95	0.89	1.02	22,787	1.09	1.07	1.10	136	1.23	0.99	1.53	98	1.27	0.98	1.64
West Midlands	2,026	1.28	1.20	1.35	38,564	1.47	1.45	1.49	117	0.99	0.79	1.25	93	1.13	0.87	1.47
East of England	1,609	0.93	0.87	0.99	26,001	0.96	0.95	0.98	150	1.20	0.97	1.48	82	0.94	0.71	1.23
London	1,775	1.05	0.99	1.12	48,171	1.21	1.19	1.22	120	0.93	0.74	1.16	96	1.09	0.84	1.41
South West	1,418	0.81	0.76	0.86	26,039	1.09	1.07	1.10	136	1.25	1.00	1.55	80	1.04	0.79	1.37
Heterogeneity p-value	<0.001				<0.001				0.005				0.043			

Table 3. Correlations between SERs by English Government Office Region for asthma mortality, asthma emergency admissions, treated asthma and severe asthma.

	Mortality	Emergency admissions	Treated asthma	Severe asthma
Mortality	1			
Emergency admissions	0.41	1		
Treated asthma	-0.38	0.25	1	
Severe asthma	0.05	0.60	0.34	1

Figure 1.Time trends in asthma mortality per million in the least deprived quintile of IMD and most deprived quintile of IMD, 3-year moving average, by broad age band.

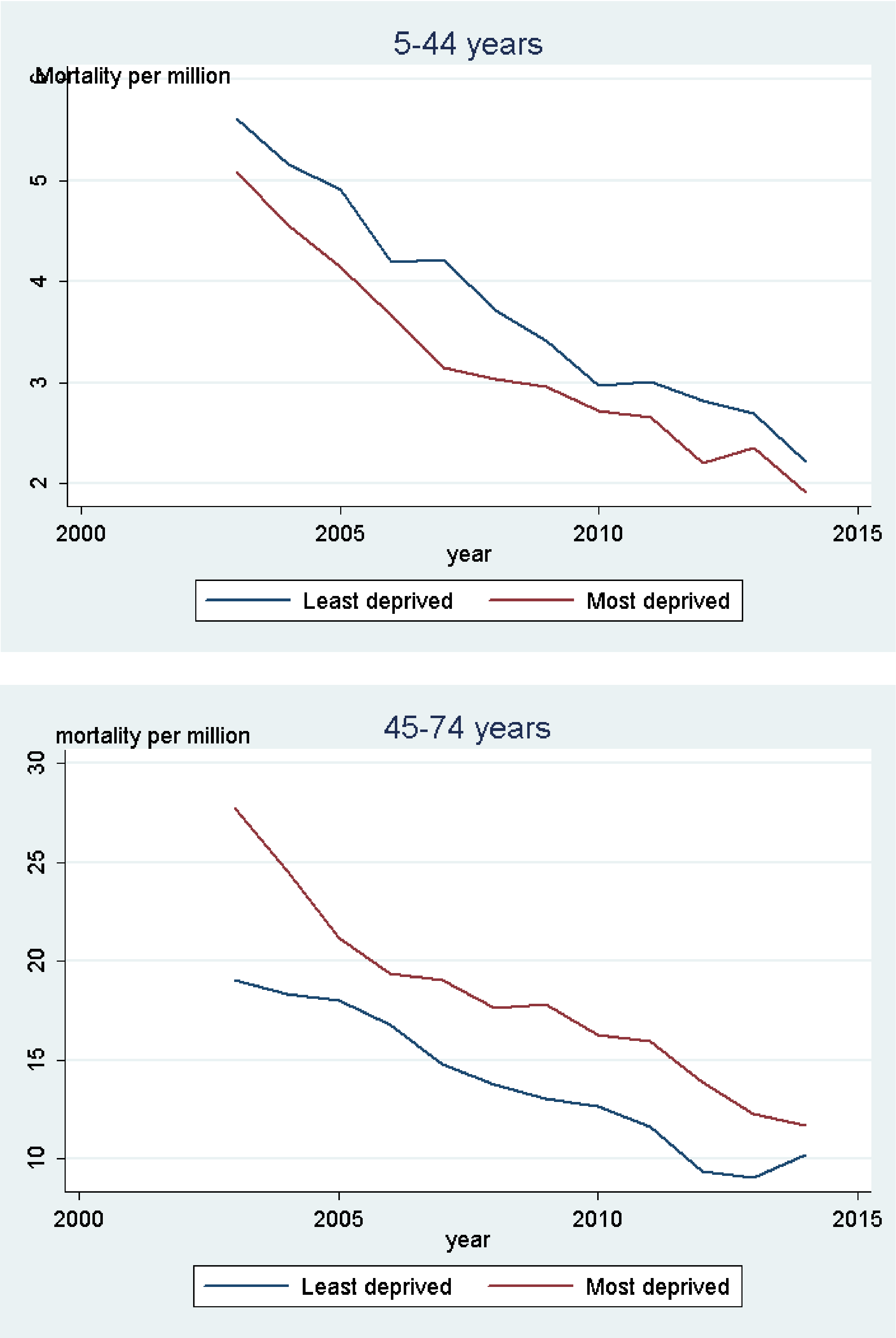

Supplement: Supplementary file 1 [file thoraxjnl-2017-210714supp001.pdf]
